# The influence of segmental and suprasegmental phonological awareness on word and pseudoword reading—A comparison between native English speakers and native Chinese speakers of English

**DOI:** 10.3389/fpsyg.2024.1214197

**Published:** 2024-02-21

**Authors:** Ying Jiang, Xiaosong Gai, Zhe Wang, Jenny Thomson

**Affiliations:** ^1^School of Psychology, Northeast Normal University, Changchun, China; ^2^Key Research Base of Humanities and Social Sciences of Higher Education in Jilin Province, Center of Mental Health Education and Research, Northeast Normal University, Changchun, China; ^3^Institute of Education and Science, Jilin Engineering Normal University, Changchun, China; ^4^School of Allied Health Professions, Nursing & Midwifery, University of Sheffield, Sheffield, United Kingdom

**Keywords:** phonological awareness, segmental and suprasegmental, word reading, pseudoword reading, second language

## Abstract

Segmental and suprasegmental phonological awareness (PA) are closely related to word reading skills in native speakers learning to read an alphabetic script as used in English. However, their roles in English word and pseudoword reading among native Chinese (NC) speakers, and how English proficiency might affect these relationships, remain less clear. This study examined the links between English segmental/suprasegmental PA and word/pseudoword reading in NC and native English (NE) speakers. Both child and adult participants were assessed on English segmental and suprasegmental PA, alongside vocabulary, at a single time point. The results showed that both segmental PA (elision and segmenting nonwords) and suprasegmental PA (aural suffix judgment and written suffix judgment) were significantly correlated with English real word and pseudoword reading of both NE and NC children, and NC adults, but not NE adults. Moreover, for NE and NC children, segmental PA correlated stronger with real word reading than suprasegmental PA after controlling for vocabulary. Among NC adults, both segmental and suprasegmental PA significantly contributed to real word reading. For pseudoword reading, after controlling for vocabulary, segmental PA had a stronger correlation among NC children and adults, while suprasegmental PA was more influential for NE children. This research gives insights into factors influencing NC speakers’ English word reading ability, bearing essential implications for enhancing second language literacy in learners from a logographic background.

## Introduction

Reading relies heavily on oral language. As Mattingly (1972) once stated, “reading is parasitic on speech” (p. 133), necessitating an understanding of both the sounds and meaning of oral language. English segmental and suprasegmental phonological awareness (PA), i.e., an ability to reflect upon and manipulate phonemes and larger units of speech, respectively, are crucial for an individual’s word reading and decoding abilities (Melby-Lervåg et al., 2012). Segmental PA involves the discernment of phonemes, which are the smallest units of sound that can differentiate meaning in a language. Suprasegmental PA encompasses the phonological attributes of language, such as stress, intonation, pitch, timing, and metre, all of which accompany segmental phonology (Thomson and Jarmulowicz, 2016). In studies with native English (NE) children, segmental and suprasegmental PA have been found to be strong predictors of word reading skills (Holliman et al., 2010; Critten et al., 2021).

However, the relevance of segmental and suprasegmental PA in the ability of NE adults to read words remains a contentious topic among scholars. Some scholars have reported significant correlations between segmental/suprasegmental PA and real word and pseudoword reading competence among NE adults (Thomson et al., 2006; Mundy and Carroll, 2012). For instance, Metsala et al. (2019) found that Canadian first-year university students’ ability to switch initial sounds in names, like “John Lennon” to “Lohn Jennon,” correlated at 0.48 with their accuracy in reading words. Other researchers argue that the influence of segmental and suprasegmental PA has less impact on later or more advanced NE speakers’ literacy performance (Torgesen, 1999; Wade-Woolley and Heggie, 2015; Yeong et al., 2017). A possible explanation is that as word reading becomes more automatic, the reliance on PA, specifically analyzing the sound structure of words, becomes less central or pivotal in the reading process.

Thus, the question arises as to how the relationships between segmental and suprasegmental PA and the ability to read words would manifest among populations with more limited English proficiency, such as native Chinese (NC) speakers. First, NC speakers’ limited immersion in English-speaking environments may constrain their English proficiency, challenging word decoding and comprehension and necessitating a stronger reliance on phonological cues when reading English words. Second, the divergent orthographic structures and phonological characteristics of Chinese and English writing systems could hinder NC speakers’ acquisition of word reading proficiency. From a segmental perspective, English is an alphabetic language wherein orthographic graphemes map to phonemes (Yeung et al., 2023). In contrast, the Chinese logographic system links written characters to meaning-bearing morphemes or multi-morpheme words, rather than to phonemes (Verhoeven and Perfetti, 2022). Additionally, Verhoeven and Perfetti note that phonemic awareness plays a varying role across different orthographies. In alphabetic languages like English, it is a significant predictor for reading, whereas in morphosyllabaries, like Chinese, its importance is not uniform. Logographic readers heavily focus on word shape and radicals that provide semantic and phonetic cues to the whole character (Chang et al., 2022). Consequently, NC speakers of English may have less phonemic processing experience in oral word reading. Moreover, scholars claim that NC speakers face challenges in phonemic production and word reading, attributed to their lack of English categorical perception abilities (Jiang et al., 2023a). These difficulties arise from the overlapping yet subtly distinct sounds between Pinyin (the official Romanization system for Mandarin) and English phonemes, which impede NC speakers’ ability to accurately perceive and process these phonemes. From a suprasegmental standpoint, English is usually regarded as stress-timed, differentiating strong and weak syllables by duration and vowel quality, whereas Chinese, being syllable-timed, lacks the equivalent of English phonological stress patterns (Liu and Takeda, 2021). This discrepancy often leads NC speakers to misplace stress in unfamiliar English words and require lots of conscious practice to master prosodic competence at the word level. If aspects of segmental PA and prosody are a challenge to acquire for NC speakers, it raises the question of whether the correlational relationships between segmental/suprasegmental PA and word reading ability are as strong, or stronger for NC speakers.

In studies with NC children, segmental and suprasegmental PA have been consistently identified as strong predictors of word reading skills (Chung et al., 2013; Wei, 2017). In their study, Deng et al. (2019) examined the relationship between PA and English word reading and reading comprehension abilities in typically developing second-grade students in Hong Kong. They assessed these abilities by measuring English segmental PA (phonemic awareness) through elision and word blending tasks, and suprasegmental PA (stress sensitivity) through tasks that targeted word, intonation, and phrase stress. This study revealed strong correlations between PA measures and both word reading and reading comprehension skills. Moreover, Jiang et al. (2023b) conducted a meta-analysis to examine the effectiveness of PA training among NC speakers. They reported that PA training significantly improved upper elementary students’ word reading abilities. These findings emphasized the pivotal role of both segmental and suprasegmental PA in enhancing English word reading ability among learners with limited English language proficiency.

However, few studies have examined the relationship between segmental and suprasegmental PA and the ability to read words in NC adults. Sun and Lee (2021) tested 74 Chinese first-year university students and revealed that segmental PA was a significant predictor of pseudoword reading ability. Yeong et al. (2017) examined the phonological processing skills of NC university international students in Australia. This included segmental PA, assessed using nonword segmentation and phoneme reversal subtests, as well as a silent phonological choice task where participants identified the nonword that sounded most like a real English word from a set on the screen via button press. Their findings indicated a moderate correlation between these phonological skills and the students’ competence in reading words. Moreover, a meta-analysis by Pan et al. (2019) demonstrated a moderate relationship (*r* = 0.41) between PA and reading performance at various levels (word, sentence, text) among NC adults. However, this analysis did not encompass suprasegmental PA, thus leaving its impact on word reading ability in NC adults unclear. To date, the only study to probe this aspect was conducted by Chung and Jarmulowicz (2017), who found that the relationship between suprasegmental PA and word reading in Taiwanese university students depended on the morphological stress patterns of derivational suffixes and the task type—receptive or productive. Notably, the ability to judge stress patterns in English derivational morphology, particularly in non-neutral derivations where stress shifts to the preceding syllable, was a predictor of word reading proficiency after controlling for short-term memory and vocabulary. Conversely, proficiency in the productive task of articulating words with neutral derivational suffixes, which maintain the base word’s stress pattern, predicted real word reading and pseudoword reading abilities. Memory and vocabulary abilities were considered, but segmental PA was not assessed in this study. As a result, it has been unclear whether speech rhythm sensitivity is related to NC adults’ word reading proficiency independent of segmental PA. This limitation is the topic of the current study.

Although PA is a strong predictor of word reading ability, it is not the exclusive explanatory factor in word reading proficiency. The Lexical Restructuring Hypothesis proposes that vocabulary expansion is crucial for promoting phonemic awareness, a pivotal element in the early stages of reading development (Metsala and Walley, 1998). This is further substantiated by the empirical findings. For example, researchers have demonstrated a significant correlation between NE children’s vocabulary and their competence in word reading (Holliman et al., 2010; Critten et al., 2021). Therefore, when attempting to predict word reading ability, it is necessary to consider vocabulary level as a control variable.

Consequently, two research questions are posed. First, how are segmental and suprasegmental PA related to English word reading abilities in NE and NC speakers, and are these relations influenced by the speakers’ level of English proficiency? Second, can segmental and suprasegmental PA predict additional variance in real word reading and pseudoword reading abilities among NE and NC speakers, after controlling for vocabulary?

The current study introduces three key innovations. Firstly, it focuses on typically-developing NE and NC populations. Typical NE speakers, representing mature English proficiency, primarily serve as a comparative sample alongside the NC groups. Chinese, recognized as a writing system with deep orthography (Zhou et al., 2018), may pose greater challenges for NC speakers in English reading, attributed to its significant orthographic difference from the alphabetic nature of English. This difficulty is in contrast to English learners from alphabetic backgrounds, such as Korean, German, or Italian, or even those from non-alphabetic but partially phonetic systems like Japanese, which uses Kana scripts (Verhoeven and Perfetti, 2022). We hypothesize that this orthographic difference significantly heightens the role of PA in English word reading for NC speakers, a proposition, if confirmed, could reshape second language teaching methodologies. Secondly, this study broadens the investigative lens beyond the frequently researched child learners to include adult word reading. Although the relevance of segmental and suprasegmental PA in learning to read for children has been established, its influence on adults has not been thoroughly investigated. Additionally, this study directly compares NE and NC learners across both child and adult groups, assessing how language background and English proficiency influence the relationship between PA and word reading ability. We posit that NE adults have already achieved a high level of proficiency in PA and word reading, thus potentially reaching what is termed a “ceiling effect.” This ceiling effect suggests that these tasks fail to elicit any variability. As a result, the contribution of PA to word reading is less evident in NE adults when compared to NC speakers and younger NE learners. Should PA be shown to independently contribute to word reading among NC speakers of English, it would advocate for the inclusion of PA training in Chinese English-language curricula. This would be the contribution of this article. Thirdly, the study broadens the scope from segmental to suprasegmental PA, providing a more comprehensive understanding of how both aspects contribute to English word reading development in NE and NC populations. If suprasegmental PA plays the same vital role in second language learners’ reading words ability, it should be emphasized in future instruction.

## Methods

### Participants

Fifty NE adults (26 females and 24 males) and one hundred NC adults (52 females and 48 males) were recruited online. All participants had no history of hearing, speech, language, or literacy difficulties, and were between 18 and 30 years old, with the NE adults having an average age of 21.60 years (SD = 2.942) and the NC adults having an average age of 22.17 years (SD = 2.712). An independent *t* test showed that there was no age difference between the two groups, *t*(147) = −1.245, *p* = 0.215. Both groups of adults were from universities of comparable academic prestige in their respective countries. British participants were from the University of Sheffield (44%) and other UK Russell Group universities (56%), while Chinese participants were from Northeast Normal University (55%) or other universities titled “Double First-Class” universities, and the First-Class disciplines in mainland China (45%). NE adults had lived in the UK from birth, used English as their only language without exposure to any other languages at home, and self-identified as NE speakers. NC adults had lived in China from birth, learnt English as their second language and had no experience of being abroad.

With parental permission, thirty NE children (17 girls and 13 boys) and thirty NC children (17 girls and 13 boys) were recruited online. All participants had no history of hearing, speech, language, or literacy difficulties, and were between 6 and 9 years old, with the NE children having an average age of 7.56 years (SD = 1.05) and the NC children having an average age of 7.45 years (SD = 0.93). An independent *t* test showed that there was no age difference between the two groups, *t*(58) = 0.440, *p* = 0.662. All NC children were raised in China, learning English as a second language, and having no experience of being abroad. But NE children have more diverse backgrounds: 37 % of NE children resided in native English-speaking countries (Britain, Canada, and America), while 63% lived in China. These children were identified by their parents as NE speakers, as English was mainly used at home and in their daily life. Parental education levels, measured in years of education, were 17 years (SD = 1.930) for NE children and 16.6 years (SD = 2.236) for NC children. An independent *t* test revealed that there was no significant difference in the parental education levels between the two groups, *t*(58) = 0.742, *p* = 0.461.

### Measurements

#### Segmental PA

Segmental PA was measured with two subtests, Elision and Segmenting Nonwords, from the CTOPP (Wagner et al., 1999). The Elision subtest requires participants to listen to a word, repeat it, and then produce the resultant phonemic sequence after omitting specified phonological elements. The Segmenting Nonword subtest requires participants to listen to a nonword, break it into its constituent phonemes, and verbalize each phoneme sequentially. There were 20 items for each. For each correct answer, one point is awarded. Cronbach’s alpha reliability coefficient for the elision and segmenting nonword tasks, as reported in the manual, are 0.87 and 0.88, respectively. In the current sample, these values are 0.92 and 0.90.

#### Suprasegmental PA

Suprasegmental PA was measured with two subtests: the aural suffix judgment test and the written suffix judgment test, created by Wade-Woolley and Heggie (2015). The aural suffix judgment test evaluates how well participants identify the correct stress in multisyllabic pseudowords, a process governed by the morphological rules of stress placement in English. Each set of stimuli consisted of a pseudoword stem (e.g., *FROsure*), a sentence that included the same pseudoword stem (e.g., The coffee has *FROsure*), and two sentences that appeared at the end of the set. In the last two sentences, the same pseudowords were pronounced in two ways (e.g., *It is FROsureful*. *It is froSUREful*). Participants were asked to choose which of these pseudowords sounded better. The test consisted of 30 items, half of which included derived pseudowords with a stress-neutral suffix (*−er, −ly, −ful, −less, −ment, −ness, −ize*) and half of which included derived pseudowords with a nonneutral suffix (*−ic, −ity, −tion*). Neutral suffixes do not change the stress pattern of a word (e.g., −*ful: FROsure, FROsureful*), but nonneutral suffixes did (e.g., −icity, *NOCtic, nocTICity*). Participants got one point if they accurately identified the stress assignment of the polysyllabic pseudoword in terms of the suffix form (e.g., It is *FROsureful*). There were two practice items prior to beginning the task. The reported reliability coefficient of Cronbach’s alpha is 0.72, and for the current sample, it is 0.60.

The written suffix judgment test examines whether participants can correctly produce the stress of a polysyllabic pseudoword based on its suffix. In this study, a computer-based test was conducted, with the experimenter sharing her computer screen with participants through Google Meet or Tencent Meeting. Participants were instructed not to use headphones, ensuring synchronous auditory reception of both stimuli and verbal communications, essential for smooth execution and real-time feedback. The test consisted of two lists of pseudowords, half of which included derived pseudowords with a stress-neutral suffix (*−er, −ly, −ful, −less, −ment, −ness, −ize*) and half of which included derived pseudowords with a nonneutral suffix (*−ic, −ity, −ion*). The first list consisted of 30 pseudoword stems (e.g., chosure). The second list was made up of 30 pseudowords derived from the pseudowords on the first list by adding suffixes (e.g., chosureful). Participants were asked to read aloud 30 pairs of pseudowords from the two lists (e.g., chosure, chosureless) from top to bottom on the computer screen. After the participants had read the first 20 pairs shown on the screen, the experimenter turned the page with a mouse while the screen showed the remaining 10 pairs. They got one point if they accurately identified the stress assignment of the polysyllabic pseudoword in terms of the suffix form. There were two practice items prior to beginning the task. The reported reliability coefficient of Cronbach’s alpha is 0.80, and for the current sample, it is 0.95.

Notably, these nonwords tasks were chosen because they help assess stress placement in pseudowords without relying on lexical recall, instead testing the application of morphological rules to new linguistic forms. Using nonwords isolates knowledge of stress patterns from word memory, tapping into participants’ implicit understanding of English phonology.

#### Word reading

The two subtests of the Test of Word Reading Efficiency (TOWRE: Torgesen et al., 1999)—Sight Word Efficiency and Phonemic Decoding Efficiency—were adapted for computer presentation. The Sight Word Efficiency test included 104 words, totaling a maximum score of 104 points, and the Phonemic Decoding Efficiency test had 63 pseudowords, with a maximum score of 63 points. Participants can clearly see all the words/pseudowords. They were asked to read as many words/pseudowords from separate lists as quickly as possible, within 45 s for each list. A practice test was given first to ensure that the participants understood the instructions. Twenty percent of the reading tests were assessed by the experimenter and an English university teacher (an NE speaker) to reduce experimenter bias. The Pearson’s correlation showed that the correlation between the two scorers was strong for real word reading (*r* = 0.92) and pseudoword reading (*r* = 0.90). This indicated consistent and correct scoring by the experimenter. Hence, the experimenter’s scoring was adopted. The split-half reliability scores reported in the manual for the TOWRE are between 0.82 and 0.97. The Cronbach’s alpha reliability coefficients in the present sample are 0.99 for Sight Word Efficiency and 0.98 for Phonemic Decoding Efficiency.

#### Vocabulary

Receptive vocabulary was measured using the British Picture Vocabulary Scale-II (BPVS-II: Dunn et al., 1997). It was adapted for computer presentation. BPVS-II is composed of 14 sets of 12 items each, totaling 168 words. Participants were asked to indicate which of four pictures shown on the computer screen best represented a word provided orally by the tester. One point is awarded for each correct answer. The reported reliability coefficient of Cronbach’s alpha is 0.94, and in the present sample, it is 0.99.

### Procedure

The study was approved by the Ethics Committee of the Division of Human Communication Sciences, the Health Sciences School of the University of Sheffield, and the Ethics Committee of the School of Psychology at Northeast Normal University. Parents of child participants completed online consent forms.

NE and NC participants were recruited online through convenience sampling by sending emails to volunteer mailing lists and student societies and posting on social media. The assessments were administered online by the experimenter (the first author) with each participant in a one-on-one context. Before starting the experiment, the adult participants received audio files from the experimenter. Every test was associated with an independent audio file. These stimuli had been recorded in a quiet space by a 43-year-old female British elementary school teacher and doctoral student specializing in Speech and Language Therapy, with a Southern British accent.

During the assessment, the experimenter shared her computer screen to present the test names, visual words, or pictures. Adult participants viewed the content on their individual computers, played each file on their mobile phones in the given sequence, and orally answered each corresponding test question. It was decided that participants would not use headphones to listen to stimuli so that the experimenter could also hear the recordings and ensure the correct stimuli were being played. For NE and NC children, a more efficient and convenient online testing method was used. This approach did not require sending audio materials to the children’s parents in advance, nor did it necessitate parental assistance in manually playing the recordings for the children. The experimenter used the screen sharing and system sound sharing features to sequentially play the audio files on the computer. Consequently, the children were able to clearly receive the sound stimuli through their own computers and respond orally. All adult and child participants completed the measures in the following order: aural suffix judgment test, elision test, segmenting non-words test, sight word efficiency test, phonemic decoding efficiency test, vocabulary test, and written suffix judgment test. The two suprasegmental PA tests were interspersed with other tests to reduce interference between the two tests. The tests took up to one hour per participant. The whole process was recorded for scoring.

### Design and data analysis

A cross-sectional correlational design was used to investigate the relative contributions of segmental and suprasegmental PA on word and pseudoword reading in NE and NC children and adults. Criterion variables were real word and pseudoword reading. Predicting variables included segmental PA (elision and segmenting nonwords) and suprasegmental PA (aural suffix judgment and written suffix judgment). Age and vocabulary were control variables.

Prior to data analysis, both univariate and multivariate outlier analyses were conducted for each measure. Univariate outliers were identified as z-scores exceeded ±3.29 (*p* < 0.001, two tailed; Tabachnick and Fidell, 2013). Multivariate outliers were identified using the Mahalanobis’ distances with *p* < 0.001. We screened all measures across four groups for normal distributions and homogeneity of variances. Normality for each measure was based on the Satorra-Bentler scaled statistics, as recommended by West et al. (1995), with skewness and kurtosis thresholds set at 2 and 7, respectively. Variance homogeneity was assessed using the Levene test. All metrics were analyzed using raw scores.

First, we calculated the descriptive statistics of all the tasks. Then, a Multivariate Analysis of Variance (MANOVA), controlling for age, was conducted to compare multiple competencies between NC and NE children, as well as adults. This MANOVA entered vocabulary, segmental PA (elision and segmenting nonwords), suprasegmental PA (aural suffix judgment and written suffix judgment), word and pseudoword reading scores simultaneously as dependent variables.

Second, we ran a Pearson’s correlational analysis to evaluate the common variance across all measures and groups. We separately examined and presented how segmental and suprasegmental PA relate to word and pseudoword reading abilities in NE and NC speakers, both children and adults. This aimed to elucidate the nature of these correlations and to determine if the English proficiency level among the speakers influenced these relationships.

Finally, hierarchical linear regression analyses were used to examine how segmental and suprasegmental PA independently contributed to real and pseudoword reading across populations, controlling for vocabulary. We used a composite score for segmental and suprasegmental PA, respectively, to determine which phonological skill contributed more to participants’ real word and pseudoword reading. The composite score for segmental PA was the mean score of the elision and segmenting nonwords tests. The suprasegmental PA used the mean score of the aural judgment and the written judgment tests. We separately ran three-step fixed-entry hierarchical regression analyses for NE children, NC children, and NC adults. The control variable (vocabulary), and the predicting variables (segmental PA and suprasegmental PA) were entered hierarchically. Initially, vocabulary was entered, followed by segmental PA or suprasegmental PA. To investigate their respective impacts on English word and pseudoword reading, we reversed the order of segmental and suprasegmental PA in the subsequent steps across two models.

## Results

The descriptive statistics of the tasks are presented in Table 1. Univariate outlier analysis led to the exclusion of one NC adult from the vocabulary task, one NC adult from the written suffix judgment task and one NE adult from the pseudoword reading task. Thus, the study included 207 participants: 30 NE children, 30 NC children, 49 NE adults and 98 NC adults. The measures across four groups showed normal distributions. The Levene test indicated homogeneity of variances (*ps* > 0.05) in adults’ aural suffix judgment and real word reading measures, and children’s elision, aural suffix judgment, and real word reading measures. Where variance homogeneity assumptions were not met, Brown–Forsythe and Welch tests were used for group comparisons. This was the case for adults’ vocabulary, elision, segmenting nonwords, written suffix judgment, and pseudoword reading measures, and to children’s vocabulary, written suffix judgment, segmenting nonwords, and pseudoword reading measures.

**Table 1:** Group comparisons on all measures in children and adults (raw scores).

Group	Variables	Hierarchy	Tasks (maximum score)	NE		NC		*F*
				Mean	SD	Mean	SD	
Children	Predictors	Control measures	Age (years)	7.56	1.05	7.45	0.93	0.440
			Vocabulary (168)	85.67	22.267	15.93	10.831	237.933***
		PA: segmental PA	Elision (20)	9.77	5.077	3.60	3.962	27.511***
			Segmenting nonwords (20)	7.00	4.185	2.73	2.288	24.002***
		PA: suprasegmental PA	Aural suffix judgment (30)	17.03	3.068	14.90	2.808	7.893**
			Written suffix judgment (30)	8.43	8.080	1.53	2.488	19.983***
	Criterion	Reading: English real word reading	Real word reading (104)	37.70	21.931	14.10	16.583	22.103***
		Reading: English pseudoword reading	Pseudoword reading (63)	17.50	11.337	5.60	6.246	25.357***
Adults	Predictors	Control measures	Age (years)	21.51	2.902	22.22	2.711	−1.471
			Vocabulary (168)	160.41	5.192	51.67	20.168	1374.388***
		PA: segmental PA	Elision (20)	16.16	3.287	8.85	4.165	115.176***
			Segmenting nonwords (20)	13.78	3.880	5.89	2.632	211.361***
		PA: suprasegmental PA	Aural suffix judgment (30)	22.14	3.824	18.40	3.148	39.933***
			Written suffix judgment (30)	26.86	2.151	18.01	3.310	288.509***
	Criterion	Reading: English real word reading	Real word reading (104)	89.88	8.894	56.55	8.396	494.646***
		Reading: English pseudoword reading	Pseudoword reading (63)	56.14	4.650	26.58	8.660	497.989***

*** *p* < 0.001, ** *p* < 0.01. Number of participants: NE children (*n* = 30), NC children (*n* = 30), NE adults (*n* = 49), NC adults (*n* = 98). For Age in years, *t*-test was reported. *F*-test was conducted on all measures.

The MANOVA comparing NE and NC children on the elision, aural suffix judgment and real word reading measures revealed significant differences, with Wilks’ lambda (*λ*) = 0.549, *F* (1, 58) = 15.060, *p* < 0.001, $\eta_{p}^{2}$ = 0.451. Additionally, the Brown–Forsythe and Welch tests showed that the differences between NE and NC children in vocabulary, written suffix judgment, segmenting nonwords, pseudoword reading tests were significant, *ps* < 0.01. NE children greatly outperformed their age-matched NC peers in all evaluated tasks, *ps* < 0.01, see Table 1. Likewise, the MANOVA comparing NE and NC adults on the aural suffix judgment and real word reading measures was also significant, with Wilks’ lambda (*λ*) = 0.225, *F* (1, 145) = 247.730, *p* < 0.001, $\eta_{p}^{2}$ = 0.775. The Brown–Forsythe and Welch tests showed that the differences between NE and NC adults in vocabulary, elision, segmenting nonwords, written suffix judgment, pseudoword reading tests were significant, *ps* < 0.001. NE adults performed significantly better than NC adults in all the tasks, *ps* < 0.001.

### Relationship between segmental and suprasegmental PA and word and pseudoword reading

Pearson’s correlations among all variables are shown in Table 2. Most predictor variables (segmental and suprasegmental PA) were significantly correlated with both reading outcome measures NE and NC children, as well as for NC adults. The control variable (vocabulary) significantly correlated with both real word and pseudoword reading abilities across four groups, with the exception of pseudoword reading in the NE child group.

**Table 2:** Pearson’s correlations among all variables.

Group	Variables	1	2	3	4	5	6	7
Children	1. Vocabulary	–	0.652***	0.567**	**0.143**	0.782***	0.817***	0.834***
	2. Elision	0.347	–	0.678***	**0.073**	0.724***	0.785***	0.745***
	3. Segmenting nonwords	0.430*	0.644***	–	0.378*	0.652***	0.635***	0.549**
	4. Aural suffix judgment	−0.035	0.194	0.232	–	**0.226**	**0.116**	**0.1**
	5. Written suffix judgment	0.223	0.751***	0.460*	0.339	–	0.870***	0.852***
	6. Real word reading	0.427*	0.649***	0.565**	0.112	0.772***	–	0.942***
	7. Pseudoword reading	0.309	0.606***	0.475**	0.231	0.804***	0.875***	–
Adults	1. Vocabulary	–	0.445***	0.340**	0.460***	0.427***	0.451***	0.376***
	2. Elision	0.232	–	0.396***	0.239*	0.429***	0.398**	0.495***
	3. Segmenting nonwords	0.101	0.449**	–	0.218*	0.382***	0.341***	0.404***
	4. Aural suffix judgment	0.137	0.006	0.230	–	0.275**	0.359***	0.262**
	5. Written suffix judgment	0.151	0.154	0.083	0.281	–	0.410***	0.412***
	6. Real word reading	0.422*	−0.046	−0.265	−0.066	−0.004	–	0.614***
	7. Pseudoword reading	0.375*	0.225	0.172	0.045	−0.092	0.474***	–

*** *p* < 0.001, ** *p* < 0.01, * *p* < 0.05. For both adults and children: bottom left, unbolded: NE group; top right, bolded: NC group.

Table 3 shows Pearson’s correlations between segmental/suprasegmental PA and word/pseudoword reading in both NE and NC children and NC adult groups. Segmental PA, including elision and nonword segmenting, was significantly correlated with real word and pseudoword reading skills in NC and NE children, but not in NE adults. Aural suffix judgment, an aspect of suprasegmental PA, correlated only with real word and pseudoword reading skills in NC adults, and not with those in either NE or NC children’s groups. However, written suffix judgment in suprasegmental PA showed significant correlations with real word and pseudoword reading skills in both NE and NC children and NC adults.

**Table 3:** Correlation between reading and phonological-related tasks in NE and NC.

Group	PA	Tasks	Real word reading	Pseudoword reading
NE children	Segmental	Elision	0.649***	0.606***
		Segmenting nonwords	0.565**	0.475**
	Suprasegmental	Aural suffix judgment	0.112	0.231
		Written suffix judgment	0.772***	0.804***
NC children	Segmental	Elision	0.785***	0.745***
		Segmenting nonwords	0.635***	0.549**
	Suprasegmental	Aural suffix judgment	0.116	0.2
		Written suffix judgment	0.870***	0.852***
NE adults	Segmental	Elision	−0.046	0.225
		Segmenting nonwords	−0.265	0.172
	Suprasegmental	Aural suffix judgment	−0.066	−0.004
		Written suffix judgment	0.045	−0.092
NC adults	Segmental	Elision	0.398**	0.495***
		Segmenting nonwords	0.341***	0.404***
	Suprasegmental	Aural suffix judgment	0.359***	0.262**
		Written suffix judgment	0.410***	0.412***

*** *p* < 0.001, ** *p* < 0.01, * *p* < 0.05.

### Independent contribution of segmental and suprasegmental PA to word and pseudoword reading

The hierarchical linear regression analyses revealed divergent influences of PA, contingent upon the age and native language background of the participants. As Table 4 presents, for NE children, all variables explained 58.3% of the variance in real word reading and 60.2% in pseudoword reading. Segmental PA significantly explained 34.2% of the variance in real word reading after accounting for English vocabulary, and an additional 6.9% when entered after suprasegmental PA. In contrast, for pseudoword reading, while segmental PA initially explained a significant 38.6% variance beyond vocabulary, this contribution diminished if suprasegmental PA was entered first, underscoring its greater relative importance for pseudoword reading among NE children.

**Table 4:** Hierarchical regressions predicting real word reading and pseudoword reading.

Group	Step	Real word reading				Pseudoword reading			
		β	*t*	*R* ^2^	*R*^2^ change	β	*t*	*R* ^2^	*R*^2^ change
NE children	Step1 vocabulary	0.217	1.522	0.183	0.183*	0.112	0.803	0.099	0.099+
	Step2 segmental PA	0.306	2.076*	0.525	0.342***	0.334	1.793+	0.485	0.386***
	Step3 suprasegmental PA	0.333	1.905+	0.583	0.058+	0.470	2.755*	0.602	0.116*
	Step2 suprasegmental PA	0.333	1.905+	0.514	0.331***	0.470	2.755*	0.552	0.453***
	Step3 segmental PA	0.396	2.076*	0.583	0.069*	0.334	1.793+	0.602	0.049+
NC children	Step1 vocabulary	0.430	3.624**	0.631	0.631***	0.601	5.509***	0.743	0.743***
	Step2 segmental PA	0.460	3.759**	0.783	0.152***	0.329	2.927**	0.821	0.078**
	Step3 suprasegmental PA	0.140	1.321	0.797	0.014	0.101	1.043	0.828	0.007
	Step2 suprasegmental PA	0.140	1.321	0.686	0.055*	0.101	1.043	0.772	0.029+
	Step3 segmental PA	0.460	3.759**	0.797	0.110**	0.329	2.927**	0.828	0.057**
NC adults	Step1 vocabulary	0.197	1.835+	0.204	0.204***	0.073	0.695	0.141	0.141***
	Step2 segmental PA	0.223	2.198*	0.272	0.068**	0.422	4.208***	0.313	0.172***
	Step3 suprasegmental PA	0.266	2.483*	0.316	0.045*	0.181	1.715+	0.334	0.021+
	Step2 suprasegmental PA	0.266	2.483*	0.281	0.078**	0.181	1.715+	0.209	0.067**
	Step3 segmental PA	0.223	2.198*	0.316	0.035*	0.422	4.208***	0.334	0.125***

*** *p* < 0.001, ** *p* < 0.01, * *p* < 0.05, + *p* < 0.1.

NC children exhibited a higher variance explanation of 79.7% in real word reading and 82.8% in pseudoword reading. Segmental PA’s initial entry accounted for 15.2% of the variance in real word reading and 7.8% in pseudoword reading, beyond vocabulary. When segmental PA was entered after suprasegmental PA, it still revealed additional variance contributions of 11% in real word reading and 5.7% in pseudoword reading.

For NC adults, the total explained variance was comparatively lower, at 31.6% for real word reading and 33.4% for pseudoword reading. Both segmental and suprasegmental PA are significant contributors to real word reading after vocabulary had been accounted for. Specifically, segmental PA explained an additional 6.8% of the variance when entered before suprasegmental PA, and an additional 3.5% when entered after it. Conversely, suprasegmental PA accounts for 7.8% additional variance when entered first, and 4.5% additional variance when following segmental PA. In pseudoword reading, segmental PA explained an additional 17.2% of variance after vocabulary when entered first, and even when entered after suprasegmental PA, it still accounted for an additional 12.5% of variance.

## Discussion

The present study extended the focus to the role of segmental PA in word reading to suprasegmental PA. First, it examined whether English proficiency influences the relationship between English segmental and suprasegmental PA and word reading ability by comparing four groups, namely NE children, NE adults, NC children, and NC adults. Second, it investigated the relative contributions of segmental as well as suprasegmental PA to NE and NC speakers’ ability to read words after controlling for English vocabulary competence. The study showed English proficiency affects how PA relates to word reading, with segmental and suprasegmental PA’s impact differing by participant age and language background.

### English proficiency influences the relationship between PA and word reading

Previous studies have extensively examined the relationship between PA and word reading among NE speakers, identifying PA as a strong predictor of their word reading proficiency. However, the link between segmental and suprasegmental PA and word reading in NC speakers of English, especially adults, is less established. This study investigates these relationships across ages and native language backgrounds.

One key finding of the study is that both segmental and suprasegmental PA were significantly correlated with the reading of real words and pseudowords in NE and NC children and NC adults. However, this correlation was not observed in NE adults. It indicates that language proficiency may be a crucial factor that influences the relationship between PA and word reading. For NE and NC children, both PA proficiency and word reading skills are generally lower than those of adults. As a result, they tend to depend more on their PA when performing word reading tasks. In contrast, NE adults usually have high English proficiency, which might lead to a ceiling effect in word reading abilities. This was evidenced by the high accuracy rates in word reading and PA tests among NE adults in this study, indicating a potentially weaker correlation between PA and word reading for this group. NC adults, despite their experiences in English language learning such as vocabulary growth and improved word reading and comprehension skills, still have not achieved full English proficiency, as indicated by the scores in Table 1. Hence, during word reading tasks, they are more reliant on prosodic cues and grapheme-phoneme conversion rules due to the ongoing development of their reading automaticity.

These outcomes can be explained through both theoretical and empirical studies. Theoretically, in accordance with Frith’s theory of reading developmental stages, individuals proceed through three stages to become skilled readers: linguistic, alphabetic, and orthographic (Frith, 1985). In the alphabetic stage, PA skills and grapheme and phoneme conversion rules are consolidated, whilst during the orthographic stage, word recognition becomes more automatic and holistic, based on larger orthographic units. Thus, whilst phonological skills are important in the earlier stages of reading, orthographic facility becomes important in more advanced stages. This is the reason why, in our study, we have observed a stronger correlation between PA and word reading among populations with lower English proficiency, who tend to depend more heavily on phonological processing skills when reading words. In contrast, proficient NE adult groups may have transitioned towards a preference for holistic word recognition strategies. Practically, our results are supported by Yeong et al. (2017), who found that orthographic skills were a significant predictor of English word reading for NE adults, but not for NC adults. Additionally, their research revealed that phonological processing skills predicted additional variance in word reading proficiency in NC adults, but not in NE adults.

It was also observed that suprasegmental PA as assessed by aural suffix judgment tasks did not correlate with NE and NC children’s real word and pseudoword reading skills, whereas written suffix judgment did. This discrepancy may be due to the nature of the aural stress suffix task as a forced-choice task; evidence for participants’ sensitivity to the affixation-driven stress rule came from comparing the scores to chance level performance. NC children’s scores on the aural stress suffix task (*M* = 14.9, SD = 2.81) did not significantly differ from chance level, *t* (29) = −0.195, *p* = 0.847. Although NE children’s performance on the written suffix judgment task (*M* = 17.03, SD = 3.07) was significantly greater than chance, *t* (29) = 3.630, *p* < 0.001, it was still marginally above this baseline. The results above suggest that NE children’s suprasegmental PA levels may still be developing and not fully matured. This further implies that the relationship between prosodic awareness and word reading may vary depending on the task type. Consistent with this, Wade-Woolley and Heggie (2015) found that NE adults’ performance on written suffix judgment tasks showed a significant correlation with real word and pseudoword reading, whereas aural suffix judgment tasks did not. Wade-Woolley et al. (2022) note that assessing prosodic competence remains difficult. Unlike segmental PA which is primarily focused on segmentals and has been determined to be a largely unidimensional construct (Anthony and Francis, 2005; Neumann et al., 2019), prosody uses multifaceted experimental tasks. These tasks include linguistic units such as words, syntactic phrases, utterances, and discourse, and also include diverse evaluation methods such as receptive evaluation and productive evaluation. The relational structure between various measures and experimental tasks used to characterize prosody is not yet fully understood.

Taken together, these results align with existing literature that underscores a significant correlation between English segmental/suprasegmental PA and word reading skills in NE and NC speakers (Mundy and Carroll, 2012; Chung and Jarmulowicz, 2017; Yeong et al., 2017; Metsala et al., 2019). More critically, this study elucidates the impact of English proficiency on the relationship between PA and word reading abilities across native language backgrounds. As language learners grow older, their reading skills typically improve, resulting in more automatic word recognition (Ruthruff et al., 2008). Consequently, the relationship between PA and word reading may weaken, especially when compared to children. In this case, relying solely on the research implemented with NE speakers might lead to an underestimation of the importance of PA in the word reading abilities of English as second language learners. The current findings indicate that PA continues to play a significant role in predicting word reading proficiency among NE and NC children and NC adults. This suggests that the significance of PA should not be neglected in English instructional programs for learners not yet proficient in the language.

### Relative contributions of segmental and suprasegmental PA differ in word and pseudoword reading of NE and NC speakers

This study investigated the extent to which both segmental and suprasegmental PA contribute to word reading and pseudoword reading in NE children and NE and NC adults after controlling for English vocabulary. The findings demonstrate a difference in the impact of these PA components that is dependent on the participants’ age and linguistic background.

Segmental PA was more strongly correlated with real word reading abilities than suprasegmental PA in both NE and NC children after controlling for English vocabulary levels. These findings align with the phonological processing theory, which posits that awareness and manipulation of individual phonemes (segmental PA) is essential for phonemic decoding, a foundational skill for reading in alphabetic languages like English (Wagner and Torgesen, 1987). This is consistent with prior research indicating that segmental PA is a strong predictor of word reading ability in both NE and NC children’s English reading development (Chung et al., 2013; Wei, 2017; Critten et al., 2021).

For NE children, suprasegmental PA showed a stronger correlation with pseudoword reading abilities than segmental PA, independent of English vocabulary. This might be attributed to their reliance on linguistic intuition at this developmental stage. Through exposure to their daily language environment, suprasegmental features like the rhythm and intonation of English words become internalized, constituting a crucial aspect of their linguistic proficiency. In pronouncing pseudowords, children might utilize these internalized suprasegmental patterns. This reliance on suprasegmental PA may reflect a developmental progression from segmental decoding to more holistic processing strategies that incorporate prosodic cues. Nevertheless, this outcome requires further empirical research for validation in the future.

For NC children, segmental PA correlated stronger with pseudoword reading abilities than suprasegmental PA, even after accounting for English vocabulary. NC children often have limited English learning experience and proficiency, which may result in an underdeveloped suprasegmental skill set. As the current findings show, NC children exhibit notably low levels of suprasegmental PA. This is evidenced by their performance on the aural suffix judgment task, which is at chance levels, and the written suffix judgment task, where their performance reaches floor level. Therefore, in the process of reading real words and pseudowords, they rely more heavily on segmental PA.

For NC adults, their segmental PA correlated stronger with their pseudoword reading ability than suprasegmental PA, even when controlling for vocabulary level. The potential explanation might be that phonological decoding, functioning as a self-teaching device (Share, 1995), has often been considered the *sine qua non* for successful reading acquisition. Segmental PA, especially the phoneme awareness, which requires the application of grapheme-phoneme rules to sound out and blend sounds, is highly useful for pseudoword pronunciation. The Dual Route Model supports the idea that reading involves two distinct routes: the lexical route, which directly accesses the orthographic representation of a word stored in the mental lexicon, and the non-lexical route, which uses grapheme-phoneme correspondences to sound out words (Coltheart et al., 2001). For NC adults, segmental PA is crucial for the non-lexical route, as it enables them to pronounce pseudowords by mapping graphemes onto phonemes. Moreover, this study extends the findings of Chung and Jarmulowicz (2017) by showing that, beyond English vocabulary and segmental PA, suprasegmental PA explained additional variance in word reading for NC adults. Firstly, this could potentially be attributed to the enhancement of suprasegmental abilities that arise from increased exposure to English reading and vocabulary enrichment, which in turn, facilitates the pronunciation proficiency for English real words. Secondly, the explanation for this phenomenon is related to the stress-timed nature of English (Deng et al., 2019). In English, word-level stress can help the perceptual matching process and facilitate the retrieval of words from the lexicon (Lindfield et al., 1999). Furthermore, stressed syllables in English typically feature full vowels, while unstressed syllables have reduced vowel sounds. Identifying stress patterns in English words helps NC adults understand when vowels are likely to undergo temporal and spectral reduction—manifesting as shorter, less intense, and less distinctly articulated sounds. This awareness can facilitate their ability to accurately recognize and pronounce these words. Likewise, segmental PA also significantly contributed to real word reading ability. This highlights the long-term and crucial role of segmental PA in word reading skills among NC speakers.

In summary, the current study revealed the role of both segmental and suprasegmental PA in word and pseudoword reading abilities beyond vocabulary knowledge, corroborating existing research on their essential role in reading development for both NE and NC speakers across ages (Thomson et al., 2013; Chung and Jarmulowicz, 2017; Wei, 2017; Critten et al., 2021). More importantly, these results emphasize the importance of segmental and suprasegmental PA in the development of word reading skills among English as a second language learners, especially those with limited proficiency.

### Implications and limitations

There are several implications. To begin with, the role of suprasegmental PA in NC speakers of English may easily be underestimated because, until now, the investigation into suprasegmental second language acquisition has been much less. However, suprasegmental knowledge is particularly noteworthy when investigating the relationship between phonology and literacy. Trofimovich and Baker (2006) argue that incorrect suprasegmental production may contribute to foreign accents more than incorrect segmental production. Therefore, English language instructors working with NC speakers should consider including activities that promote both segmental and suprasegmental PA to support their reading development.

Additionally, research indicates that native language and English proficiency affect how segmental and suprasegmental PA influence the ability to read words. This suggests English teaching should be tailored to individual needs. Understanding PA’s role in different native language backgrounds is crucial for improving second language instruction strategies.

Finally, much of the data linking NC speakers’ prosodic competence and reading has been based on concurrent associations up until now. Longitudinal studies are required to disentangle the role of prosodic competence across stages of reading learning in NC speakers of English.

Several limitations of this study have been noted. First, suprasegmental PA only assessed adults’ sensitivity to stress at the word level; other aspects of prosody, such as phrasing, timing, and intonation, were not explicitly assessed. Similarly, more empirical evidence is required to consolidate recent findings and see how prosodic speech sensitivity relates to other aspects of reading skills, such as reading comprehension. Incorporating multiple measures of PA and word reading could provide a more comprehensive understanding of the relationship between these skills in native and non-native English speakers.

Second, one potential limitation of the study is the small sample size, especially for the NE and NC children’s groups. This may restrict the generalizability of findings to a larger population. Future research could increase sample sizes for all groups and include participants from diverse backgrounds and regions to ensure result reliability.

Third, the BPVS-II, primarily standardized for the British population, may not sufficiently involve the linguistic nuances and cultural lexicon of non-British groups, including Chinese, Americans, and Canadians. This limitation implies a need for caution when generalizing BPVS results across diverse linguistic groups. Future research should use culturally sensitive assessments to ensure broader applicability of the findings.

Fourth, considering the online nature of our experiment, and despite the requirement for participants to have normal visual acuity as part of our recruitment criteria, the visibility of stimuli could not be rigorously controlled due to the variability in computer screen sizes and participants’ varying proximities to their screens.

Fifth, in this study, we implemented a time-limited approach for the word reading tests. For future research, it might be more beneficial to evaluate the actual word reading test based solely on “accuracy”, without the constraint of a time limit. This would allow for an assessment of the participants’ word reading proficiency with polysyllabic words. Consequently, researchers could conduct an error analysis based on these findings.

## Conclusion

This study assessed the impact of segmental and suprasegmental PA on word reading in both NE and contexts, examining the role of English proficiency. Significant correlations were found between both types of PA and the ability to read real words and pseudowords in NE and NC children, as well as NC adults, but not in NE adults. In NE and NC children, segmental PA was more closely correlated with real word reading than suprasegmental PA, even when accounting for vocabulary. Conversely, for NC adults, both segmental and suprasegmental PA independently predicted real word reading. For pseudoword reading, segmental PA demonstrated a more robust correlation among NC children and adults, while in NE children, suprasegmental PA prevailed over segmental PA. The findings supplement the theoretical evidence and facilitate the design of suitable education and language teaching programs for English as a second language learners.

## Data availability statement

The raw data supporting the conclusions of this article will be made available by the authors, without undue reservation.

## Ethics statement

The studies involving humans were approved by Ethics Committee of the School of Allied Health Professions, Nursing & Midwifery of the University of Sheffield, and the Ethics Committee of the School of Psychology at Northeast Normal University. The studies were conducted in accordance with the local legislation and institutional requirements. Written informed consent for participation in this study was provided by the participants’ legal guardians/next of kin.

## Author contributions

YJ: conceptualization, investigation, and writing, reviewing and editing. JT: conceptualization, investigation, and reviewing and editing. XG: conceptualization and reviewing. ZW: Conceptualization and reviewing. All authors contributed to the article and approved the submitted version.
